# Bisphosphonate Treatment Ameliorates Chemotherapy-Induced Bone and Muscle Abnormalities in Young Mice

**DOI:** 10.3389/fendo.2019.00809

**Published:** 2019-11-19

**Authors:** Alyson L. Essex, Fabrizio Pin, Joshua R. Huot, Lynda F. Bonewald, Lilian I. Plotkin, Andrea Bonetto

**Affiliations:** ^1^Department of Anatomy, Cell Biology & Physiology, Indiana University School of Medicine, Indianapolis, IN, United States; ^2^Department of Surgery, Indiana University School of Medicine, Indianapolis, IN, United States; ^3^Indiana Center for Musculoskeletal Health, Indiana University School of Medicine, Indianapolis, IN, United States; ^4^Simon Comprehensive Cancer Center, Indiana University, Indianapolis, IN, United States; ^5^Department of Orthopaedic Surgery, Indiana University School of Medicine, Indianapolis, IN, United States; ^6^IUPUI Center for Cachexia Research, Innovation and Therapy, Indiana University School of Medicine, Indianapolis, IN, United States; ^7^Department of Otolaryngology – Head & Neck Surgery, Indiana University School of Medicine, Indianapolis, IN, United States

**Keywords:** muscle, bone, cachexia, chemotherapy, bisphosphonates

## Abstract

Chemotherapy is frequently accompanied by several side effects, including nausea, diarrhea, anorexia and fatigue. Evidence from ours and other groups suggests that chemotherapy can also play a major role in causing not only cachexia, but also bone loss. This complicates prognosis and survival among cancer patients, affects quality of life, and can increase morbidity and mortality rates. Recent findings suggest that soluble factors released from resorbing bone directly contribute to loss of muscle mass and function secondary to metastatic cancer. However, it remains unknown whether similar mechanisms also take place following treatments with anticancer drugs. In this study, we found that young male CD2F1 mice (8-week old) treated with the chemotherapeutic agent cisplatin (2.5 mg/kg) presented marked loss of muscle and bone mass. Myotubes exposed to bone conditioned medium from cisplatin-treated mice showed severe atrophy (−33%) suggesting a bone to muscle crosstalk. To test this hypothesis, mice were administered cisplatin in combination with an antiresorptive drug to determine if preservation of bone mass has an effect on muscle mass and strength following chemotherapy treatment. Mice received cisplatin alone or combined with zoledronic acid (ZA; 5 μg/kg), a bisphosphonate routinely used for the treatment of osteoporosis. We found that cisplatin resulted in progressive loss of body weight (−25%), in line with reduced fat (−58%) and lean (−17%) mass. As expected, microCT bone histomorphometry analysis revealed significant reduction in bone mass following administration of chemotherapy, in line with reduced trabecular bone volume (BV/TV) and number (Tb.N), as well as increased trabecular separation (Tb.Sp) in the distal femur. Conversely, trabecular bone was protected when cisplatin was administered in combination with ZA. Interestingly, while the animals exposed to chemotherapy presented significant muscle wasting (~-20% vs. vehicle-treated mice), the administration of ZA in combination with cisplatin resulted in preservation of muscle mass (+12%) and strength (+42%). Altogether, these observations support our hypothesis of bone factors targeting muscle and suggest that pharmacological preservation of bone mass can benefit muscle mass and function following chemotherapy.

## Introduction

Cachexia is experienced by anywhere from 20 to 80% of cancer patients, and is ultimately responsible for poorer outcomes, increased morbidity rates and reduced chance of survival (1–3). Cachexia is frequently accompanied by several complications, such as muscle weakness, fatigue, anorexia, as well as metabolic and energy imbalances (4, 5). All these complications often lead to impaired quality of life in patients affected with cachexia, not to mention the increased economic burden (6). While the loss of lean body mass that follows the development of a tumor is frequently related with reduced responsiveness to and augmented toxicities of anticancer therapies (7, 8), we and others have shown that anticancer therapies alone are able to promote the development of cachexia (9–13).

The multisystemic and multiorgan effects of cancer and its treatments have been well described, although the mechanisms associated with these remain elusive (14). To this end, recent interest has grown in the area of the so-called “muscle-bone crosstalk,” primarily based on the idea that bone- and muscle-derived factors are able to reciprocally influence the two tissues beyond their mechanical relationship. In particular, there is mounting interest in exploring the communication between muscle and bone by means of biochemical, circulating factors (15, 16). Bone secretes soluble factors that can signal directly to skeletal muscle (17, 18). For example, Waning et al. elegantly showed that release of TGFβ from the bone matrix in a setting of bone metastases contributes to muscle weakness by decreasing Ca^2+^-induced muscle force production, thus indicating that bone-derived factors may directly affect muscle function (19).

Pathologic bone loss has been historically well documented in metastatic breast cancers and multiple myeloma and, patients undergoing treatment of a variety of tumors have been reported to be at higher risk of bone loss (20). We and others have provided evidence of a direct link between chemotherapy administration and the appearance of muscle and bone alterations consistent with a cachectic phenotype in experimental animals (13, 21). However, whether anticancer therapies promote disruption of the normal muscle-bone communication and whether preservation of bone mass can have beneficial implications on the preservation of muscle mass and strength is currently unknown.

Several bone-targeted agents, primarily bisphosphonates, were developed to stop osteoclasts from resorbing bone in order to treat pathologic conditions, such as osteoporosis and metastatic bone disruption (22). Bisphosphonates are potent antiresorptive drugs endowed with high selectivity for bone, due to their capacity to directly bind to hydroxyapatite (23). Specifically, zoledronic acid, has been tested as a bone-preserving agent in multiple diseases, including cancer (24–26). In breast cancer, zoledronic acid has been investigated for its anti-bone metastasis effects and for the potential ability to counteract tumor growth within bone (27–29). Additionally, bisphosphonate administration was used to treat skeletal events and hypercalcinemia in prostate cancer, although the potential beneficial effects of such treatment remains to be clarified (30–33). Whether bisphosphonates can also directly target muscle mass and affect muscle function remain unclear.

Interestingly, Yoon et al. showed that administration of the antiresorptive agent pamidronate to dystrophic *mdx* mice revealed positive effects on bone and muscle mass (34), although they did not provide evidence of a direct effect of bisphosphonates on muscle homeostasis. Along the same line, a clinical study showed that pediatric burn patients treated with bisphosphonates to the extent of counteracting bone resorption also present with substantial preservation of muscle mass (35). In line with previous findings (19), we recently showed that one of the mechanisms through which bisphosphonates act is likely by limiting the release of TGFβ from the bone matrix (36). The release of TGFβ prevents the activation of SMAD2/3-dependent pro-atrophy signaling in skeletal muscle, thereby suggesting that bisphosphonate administration may potentially serve as a tool for the maintenance of skeletal muscle mass in various disease states (36). These findings in conjunction with existing clinical applications suggest a potential role for zoledronic acid administration in treatment of cancer-related comorbidities, such as cachexia.

In the present study, we characterized an *in vivo* model of chemotherapy-induced cachexia in young, normal mice (37). Herein, we report the effects associated with bisphosphonate administration on the preservation of bone volume, as well as skeletal muscle mass and strength. These results provide further evidence for muscle-bone crosstalk in the pathogenesis of cachexia induced by anticancer drugs and the therapeutic potential of harnessing this cross-tissue interaction to benefit muscle mass and function following anticancer treatments by bisphosphonate administration.

## Methods

### Animals

**Figure 1:**
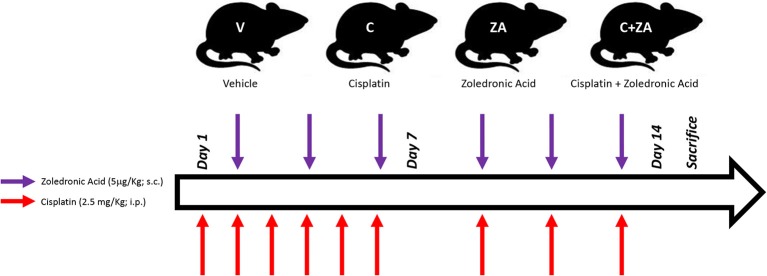
Schematic representation of the *in vivo* model. 8-week old male CD2F1 mice were exposed to i.p. cisplatin injections (C; 2.5 mg/kg), alone or in combination with zoledronic acid (ZA; 5 μg/kg), administered s.c. The control mice received equal volumes of sterile saline (V). The red arrows indicate the day of cisplatin treatment, whereas the purple arrows the day of ZA administration.

All animal experiments were conducted with the approval of the Institutional Animal Care and Use Committee at the Indiana University School of Medicine and were in compliance with the National Institutes of Health Guidelines for Use and Care of Laboratory Animals and with the ethical standards laid down in the 1964 Declaration of Helsinki and its later amendments. All animals were maintained on a regular dark-light cycle (light from 8 a.m. to 8 p.m.), with free access to food and water during the whole experimental period. Briefly, 8-week old CD2F1 male mice (Envigo, Indianapolis, IN) were used (*n* = 5–8/group). In a first set of experiments, mice were treated with vehicle (sterile saline; V) or cisplatin (2.5 mg/kg, i.p.; C) for up to 2 weeks, similar to what reported in Chen et al. (37). In another set of experiments, mice were randomized into four groups: control mice receiving vehicle alone (V), mice receiving cisplatin (C), mice treated with zoledronate (ZA), and animals receiving the combination cisplatin+ZA (C+ZA). The animals received cisplatin (2.5 mg/kg, i.p.) or ZA (5 μg/kg, s.c.), as shown in Figure 1 and in line with previously tested dosing schedules (19, 37). The mice were monitored for the entire duration of the experiments. At the time of sacrifice, no animals were excluded from the study. Several tissues were collected, weighed, snap frozen in liquid nitrogen and stored at −80°C for further analyses. The tibialis anterior muscle was frozen in liquid nitrogen-cooled isopentane, mounted in OCT and stored for morphological analyses.

### Body Composition Assessment

The quantification of lean (muscle) and fat (adipose) mass was assessed at baseline and the day before sacrifice in physically restrained mice, by means of an EchoMRI-100 (EchoMRI, Houston, USA), as previously shown (38). Data are expressed as variations over the baseline values.

### Grip Strength

The evaluation of the whole body strength in mice was assessed as previously described (39). The absolute grip strength (peak force, expressed in grams) was recorded by means of a grip strength meter (Columbus Instruments, Columbus, OH, USA). Five measurements were completed, and the top three measurements were included in the analysis. In order to avoid habituation, the animals were tested for grip strength no more than once weekly.

### Micro Computed Tomography (CT) Analysis of Femurs Bone Morphometry

MicroCT scanning was performed to measure morphological indices of metaphyseal regions of femurs. After euthanasia, the left femurs were wrapped in saline-soaked gauze and frozen at −20°C until imaging. Bone samples were rotated around their long axes and images were acquired using a Bruker Skyscan 1176 (Bruker, Kontich, Belgium) with the following parameters: pixel size = 9 μm^3^; peak tube potential = 50 kV; X-ray intensity = 500 μA; 0.3° rotation step. Calibration of the grayscale levels was performed using a hydroxyapatite phantom. Based on this calibration and the corresponding standard curve generated, the equivalent minimum calcium hydroxyapatite level was 0.42 g/cm^3^. Raw images were reconstructed using the SkyScan reconstruction software (NRecon; Bruker, Kontich, Belgium) to 3-dimensional cross-sectional image data sets using a 3-dimensional cone beam algorithm. Structural indices were calculated on reconstructed images using the Skyscan CT Analyzer software (CTAn; Bruker, Kontich, Belgium). Cortical bone was analyzed by threshold of 160–255 in the femoral mid-shaft. Cortical bone parameters included periosteal perimeter (Ps.Pm), bone area/tissue area (BA/TA), cortical thickness (Ct.Th) and cortical porosity (Ct.Po). Trabecular bone was analyzed between 1.0 and 2.0 mm under the femoral distal growth plate using a threshold of 80–255. Trabecular parameters included bone volume fraction (BV/TV), number (Tb.N), thickness (Tb.Th), separation (Tb.Sp), and pattern factor (Tb.Pf).

### Assessment of Muscle Cross Sectional Area (CSA)

Ten μm-thick cryosections of tibialis anterior muscles taken at the mid-belly were processed for immunostaining, as shown in Bonetto et al. (39). Samples were marked with a histology marking pen, blocked in phosphate buffered saline (PBS) containing 8% bovine serum albumin for 1 h at room temperature, and incubated at 4°C overnight with dystrophin primary antibody [Developmental Studies Hybridoma Bank, Iowa City, IA; #MANDRA1(7A10)] diluted in PBS. After the overnight incubation, samples were incubated with a secondary antibody (ThermoFisher Scientific; AlexaFluor 594 # A-11032) for 1 h. Samples were then washed with PBS and mounted with ProLong Antifade mounting medium (ThermoFisher Scientific). For determination of the CSA, the entire muscle section was imaged and quantified by using the Lionheart XL microscope system and the Gen5 software (BioTek, Winooski, VT).

### Cell Lines

Murine C2C12 skeletal myoblasts (ATCC, Manassas, VA) were grown in high glucose DMEM supplemented with 10% FBS, 100 U/ml penicillin, 100 mg/ml streptomycin, 100 mg/ml sodium pyruvate, 2 mM L-glutamine, and maintained at 37°C in 5% CO_2_, as shown in Pin et al. (40). Myotubes were generated by exposing the myoblasts to DMEM containing 2% horse serum (i.e., differentiation medium, DM), and replacing the medium every other day for 5 days. In order to determine the effects on myotube size dependent on bone-derived factors, myotubes were exposed to 20% bone conditioned medium (CM) for up to 48 h.

### Generation of Bone-Derived Conditioned Medium (CM)

Bone-derived CM was generated as shown in Davis et al. (41). Right femur and tibia from vehicle (V)- and cisplatin (C)-treated mice were carefully cleaned of muscle and fibrous tissues, epiphyses cut, and then marrow-flushed multiple times with αMEM. These long bones cortical preparations were then cultured *ex vivo* in 10% FBS and 1% penicillin/streptomycin (P/S)-αMEM for 48 h. CM was collected and stored at −20°C.

### Assessment of Myotube Size

C2C12 cell layers were fixed in ice-cold acetone-methanol and incubated with an anti-Myosin Heavy Chain antibody (MF-20, 1:200; Developmental Studies Hybridoma Bank, Iowa City, IA) and an AlexaFluor 488-labeled secondary antibody (Invitrogen, Grand Island, NY), as reported in Pin et al. (40). Analysis of myotube size was performed by measuring the minimum diameter of long, multi-nucleate fibers avoiding regions of clustered nuclei on a calibrated image using the Image J 1.43 software (42). Three biological replicates (*n* = 3) were generated for each experimental condition, and about 250–350 myotubes per replicate were measured. The results of each replicate were then averaged to obtain the final myotube size.

### Real-Time Quantitative PCR

Total mRNA from quadriceps muscle was isolated using the miRNeasy Mini Kit (Qiagen, Germantown, MD, USA) and following the protocol provided by the manufacturer. RNA was quantified using a Synergy H1 Spectrophotometer (BioTek Instruments, Winooski, VT, USA). RNA integrity was checked by electrophoresis on a 1.2% agarose gel containing 0.02 M morpholinopropanesulfonic acid and 18% formaldehyde. Total RNA was reverse transcribed to cDNA using the Verso cDNA Kit (Thermo Fisher Scientific). Transcript levels were measured by real-time PCR (Light Cycler 96; Roche), taking advantage of the TaqMan Gene Expression Assay System (Thermo Fisher Scientific). Expression levels for atrogin-1 (Mm00499523_m1) and MuRF-1 (Mm01185221_m1) were quantitated. Gene expression was normalized to TATA-binding protein (TBP; Mm01277042_m1) levels using the standard 2^−Δ*Ct*^ methods.

### Statistical Analysis

Results were presented as means ± SEM. Significance of the differences was determined by unpaired *t*-test when two groups were investigated. When more than two treatments were tested, two-way analysis of variance (ANOVA) followed by Tukey's multiple comparisons test were performed. The interaction *p*-value was reported exclusively when significant. Differences were considered significant when *p* < 0.05.

## Results

### Cisplatin Treatment Leads to Progressive Body Weight Loss and Muscle Depletion

**Figure 2:**
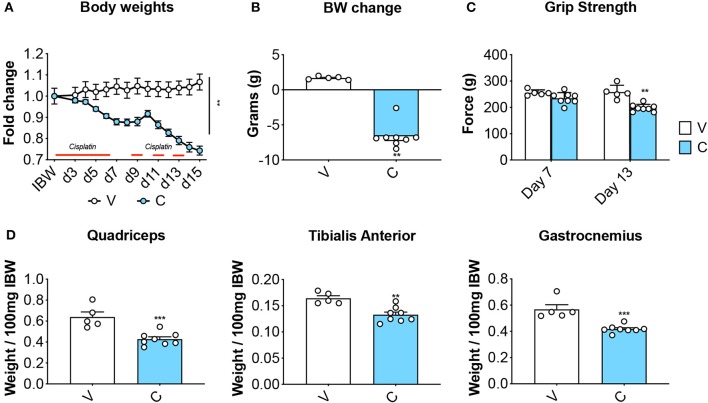
Cisplatin causes body weight loss and muscle depletion. Body weight curves **(A)**, body weight change (i.e., body weight at time of sacrifice vs. initial body weight) **(B)**, whole body grip strength (reported as peak force measured at day 7 and day 13) **(C)** and skeletal muscle weights **(D)** in mice exposed to cisplatin (*n* = 8). Control animals (V; *n* = 5) were administered equal volumes of sterile saline. Muscle weights were normalized to the Initial Body Weight (IBW) and expressed as weight/100 mg IBW. Data (means ± SEM) are expressed in grams. Significance of the differences: **p < 0.01, ***p < 0.001 vs. V.

Eight-week old CD2F1 male mice (*n* = 5) were exposed to daily cisplatin administration (C; 2.5 mg/kg, i.p.) for up to 2 weeks, while control mice (V) received equal volumes of vehicle (i.e., sterile saline). In line with previous findings (37), the animals treated with chemotherapy showed progressive body weight loss (Figure 2A), resulting in marked net loss of body weight (−6.6 g vs. initial body weight; *p* < 0.01 vs. V) (Figure 2B). In agreement with our published observations (9), the mice receiving cisplatin also showed progressive loss of skeletal muscle strength (−23% vs. V, *p* < 0.01 at day 13) (Figure 2C). These effects were consistent with marked depletion of muscle mass, as suggested by the weights of the tibialis anterior, gastrocnemius and quadriceps (Figure 2D).

### Cisplatin Treatment Leads to Severe Bone Loss

**Figure 3:**
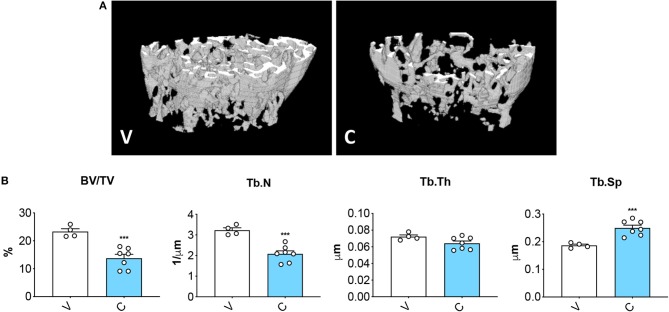
Cisplatin causes severe loss of bone mass. 3D rendering of microCT-based analysis of trabecular bone in femurs from mice receiving V (*n* = 4) or C (*n* = 7) **(A)**. Assessment of trabecular bone volume (BV/TV; expressed as %), trabecular number (Tb.N; expressed as 1/μm), trabecular thickness (Tb.Th; expressed as μm) and trabecular separation (Tb.Sp; expressed as μm) in femoral bones **(B)**. Data are reported as means ± SEM. Significance of the differences: ***p < 0.001 vs. V.

MicroCT assessment of the microarchitecture of femurs excised from mice treated with cisplatin displayed severe loss of cancellous bone (Figures 3A,B), as demonstrated by decreased trabecular bone volume ratio (BV/TV; −41%, *p* < 0.001 vs. V) and trabecular number (Tb.N; −36%, *p* < 0.001 vs. V), as well as by the increased trabecular separation (Tb.Sp; +34%, *p* < 0.05 vs. V). The data are consistent with previous evidence supporting the idea that chemotherapy administration associates with impaired bone homeostasis (13, 21).

### Myotubes Exposed to Bone Conditioned Medium (CM) From Cisplatin-Treated Mice Display Severe Atrophy

**Figure 4:**
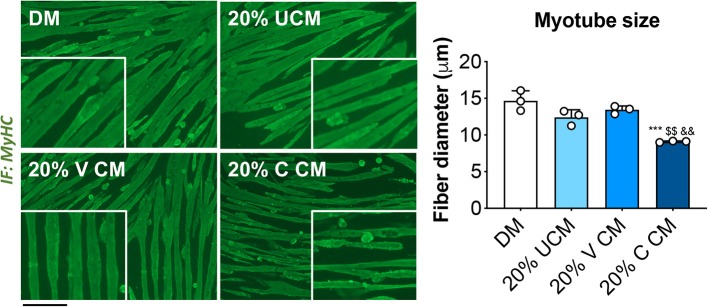
Myotubes exposed to bone conditioned medium from cisplatin-treated mice display severe atrophy. Fully differentiated (5 days) C2C12 myotubes exposed to 20% bone conditioned medium (CM) from animals treated with V or C for up to 48 h. CM was generated by incubating the bones in medium for 48 h. Controls were exposed to either normal horse serum-containing differentiation medium (DM) or 20% αMEM-containing unconditioned medium (UCM). Myotubes were stained for Myosin Heavy Chain (MyHC, green) and myotube size was measured by using the ImageJ software. 250–300 myotubes were measured, *n* = 3. Scale bar: 100 μm. Images were recorded using a 10X magnification (insert: 20X). Data are expressed as means ± SEM. Significance of the differences: ***p < 0.001 vs. DM; ^$$^*p* < 0.01 vs. UCM; ^&&^*p* < 0.01 vs. V CM.

In order to clarify whether cisplatin-induced muscle wasting was triggered by bone-derived soluble factors released upon bone destruction, we exposed fully differentiated C2C12 murine myotubes to 20% bone CM generated by incubating femora and tibiae excised from vehicle (V)- and cisplatin (C)-treated mice in αMEM-containing medium for up to 48 h. The myotubes exposed to 20% C CM displayed severe atrophy compared to V CM, as well as with respect to the myotubes cultured in normal horse serum-containing medium (DM) or unconditioned αMEM-containing (UCM) (Figure 4). These observations suggest that mediators released by bone following chemotherapy treatment may play a direct role in causing muscle fiber shrinkage.

### ZA Administration Is Unable to Counteract Cisplatin Effects on Body Weight

**Figure 5:**
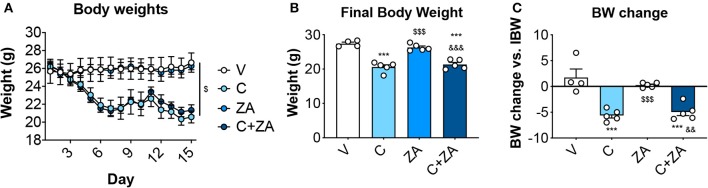
ZA fails to preserve body weight in cisplatin-treated mice. Body weight curves **(A)**, final body weight **(B)** and body weight change (i.e., body weight at time of sacrifice vs. initial body weight) **(C)** in mice exposed to C, alone or in combination with ZA (*n* = 4–5). Control animals (V) were administered equal volumes of sterile saline. Data (means ± SEM) are expressed in grams. Significance of the differences: ***p < 0.001 vs. V; ^$$$^*p* < 0.001 vs. C; ^&&^*p* < 0.01, ^&&&^*p* < 0.001 vs. ZA.

**Figure 6:**
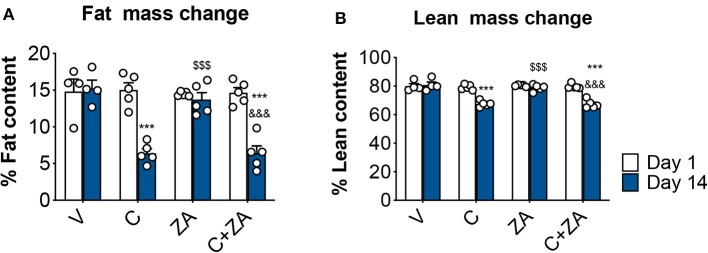
Fat and lean content in chemotherapy-treated mice is not protected by ZA administration. Fat **(A)** and lean **(B)** mass on day 1 and day 14 were assessed in mice exposed to C and/or ZA (*n* = 4–5) by using EchoMRI. Data (means ± SEM) are expressed as percentage of body mass. Significance of the differences: ***p < 0.001 vs. V; ^$$$^*p* < 0.001 vs. C; ^&&&^*p* < 0.001 vs. ZA (at the respective time point).

We then investigated whether bone preservation by bisphosphonate treatment also protects skeletal muscle mass in combination with routinely-used chemotherapy regimens. We exposed 8-week old CD2F1 male mice (*n* = 5) to cisplatin (C; 2.5 mg/Kg) (37), alone or in combination with zoledronic acid (ZA; 5 μg/Kg) (19), for up to 2 weeks (Figure 1). In line with the observations reported in Figure 2, the animals exposed to cisplatin displayed marked and progressive loss of body mass (Figure 5A), resulting in significantly reduced body weight (−25%, *p* < 0.01 vs. V) (Figures 5B,C). On the other hand, ZA administration was well tolerated and did not show evidence of toxicity, as also suggested by the absence of body weight changes compared to the V group (Figure 5). Despite this, ZA administration did not show protective effects on body mass when combined with cisplatin, reporting a body weight change of −4.96 g vs. day 1 in the animals receiving the combined treatment (*p* < 0.01 vs. V). Consistently, body composition assessment by Echo MRI revealed progressive loss of fat content (−58%, *p* < 0.001 vs. V; Figure 6A) and lean mass (−16%, *p* < 0.001 vs. V; Figure 6B) compared to day 1, whereas ZA did not show any preservation of fat and lean tissue when administered in combination with cisplatin (Figure 6). These observations were further corroborated by the observation that the gonadal adipose tissue mass was not preserved in the mice receiving cisplatin and ZA (Figure S1).

### Trabecular Bone Is Preserved in the Mice Receiving the Combination C+ZA

**Figure 7:**
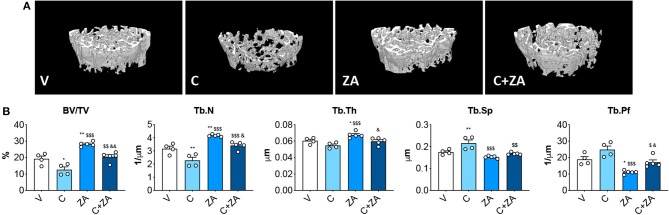
Trabecular bone is preserved in the mice administered the combination C+ZA. 3D reconstruction of microCT-based histomorphometry analysis in femurs from mice receiving V, C, ZA, and C+ZA (*n* = 4–5) **(A)**. Assessment of trabecular bone volume (BV/TV; expressed as %), trabecular thickness (Tb.Th; expressed as μm), trabecular separation (Tb.Sp; expressed as μm), trabecular number (Tb.N; expressed as 1/μm) and trabecular pattern factor (Tb.Pf; expressed as 1/μm) in femoral bones **(B)**. Data are reported as means ± SEM. Significance of the differences: *p < 0.05, **p < 0.01 vs. V; ^$$^*p* < 0.01, ^$$$^*p* < 0.001 vs. C; ^&^*p* < 0.05, ^&&^*p* < 0.01 vs. ZA.

**Figure 8:**
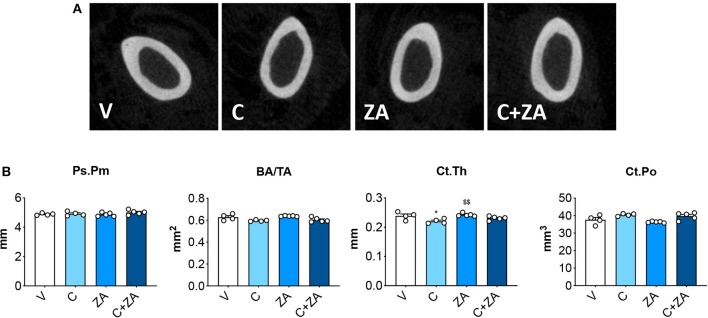
Cortical bone is minimally affected by cisplatin. 2D rendering of microCT-based histomorphometry analysis of cortical bone in femurs from mice receiving V, C, ZA and C+ZA (*n* = 4–5) **(A)**. Assessment of periosteal perimeter (Ps.Pm; expressed as mm), bone area/tissue area (BA/TA; expressed as mm^2^), cortical thickness (Ct.Th; expressed as mm) and cortical porosity (Ct.Po; expressed as mm^3^) in femoral bones **(B)**. Data are reported as means ± SEM. Significance of the differences: *p < 0.05 vs. V; ^$$^*p* < 0.01 vs. C.

microCT analysis of femoral bone from animals exposed to cisplatin revealed marked loss of cancellous bone (Figures 7A,B). We observed reduced BV/TV (−35%, *p* < 0.05 vs. V) and Tb.N (−28%, *p* < 0.05 vs. V), as well as increased Tb.Sp (+24%, *p* < 0.05 vs. V). ZA treatment alone had a beneficial effect on bone structure, as revealed by significantly elevated BV/TV (+46%, *p* < 0.01 vs. V), trabecular thickness (Tb.Th; +12%, *p* < 0.05 vs. V) and Tb.N (+32%, *p* < 0.001 vs. V), as well as by decreased Tb.Sp (−13%, *p* < 0.01 vs. V) and trabecular pattern factor (Tb.Pf; −42%, *p* < 0.01 vs. V). Interestingly, when combined with cisplatin, ZA was able to preserve bone structure, with BV/TV (+62%, *p* < 0.01 vs. C), Tb.N (+50%, *p* < 0.01 vs. C), Tb.Sp (−22%, *p* < 0.01 vs. C) and Tb.Pf (−31%, *p* < 0.05 vs. C) showing no difference with respect to the V group (Figure 7). Further, cisplatin-treated animals displayed reduced cortical thickness (−8%, *p* < 0.05 vs. V), which was substantially preserved following ZA treatment, although no other alterations in cortical bone geometry were detected (Figure 8). The loss of trabecular bone appeared milder in the animals receiving C+ZA compared to the animals treated with cisplatin alone (BV/TV: −35% in C vs. V, −27% in C+ZA vs. ZA; Tb.N: −28% in C vs. V, −18% in C+ZA vs. ZA; TB.Sp: +23% in C vs. V, +9% in C+ZA vs. ZA), although no significant interaction was observed between cisplatin and ZA based on the two-way ANOVA analysis (Figure 7). Altogether, these observations suggest that ZA does not completely counteract cisplatin-induced bone loss and that bone mass is likely maintained as a result of ZA-derived bone formation.

### Bisphosphonates Improve Muscle Size and Function in Cisplatin-Treated Animals

**Figure 9:**
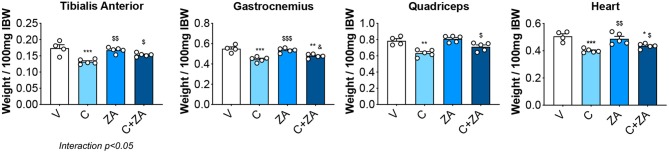
Skeletal muscle mass is partially preserved in the mice treated with C+ZA. Skeletal muscle and heart weights in mice administered C, alone or combined with ZA (*n* = 4–5). Weights were normalized to the Initial Body Weight (IBW) and expressed as weight/100 mg IBW. Data are expressed as means ± SEM. Significance of the differences: *p < 0.05, **p < 0.01, ***p < 0.001 vs. V; ^$^*p* < 0.05, ^$$^*p* < 0.01, ^$$$^*p* < 0.001 vs. C; ^&^*p* < 0.05 vs. ZA.

**Figure 10:**
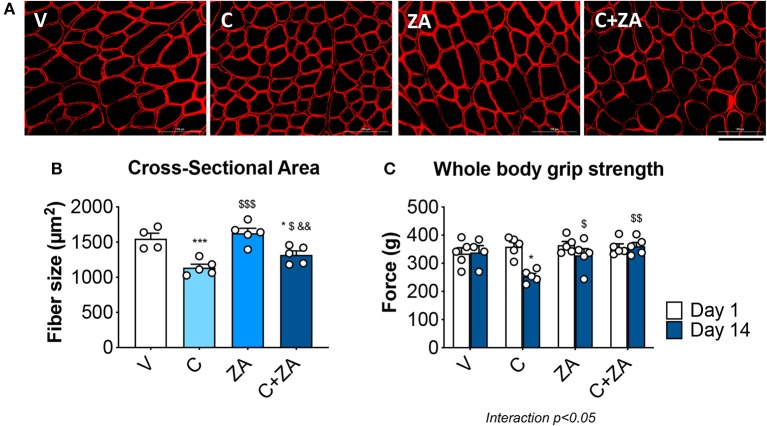
Cisplatin-induced muscle weakness is counteracted by ZA administration. Representative images of immunofluorescence staining for dystrophin **(A)** and quantification of the cross-sectional area **(B)** in the tibialis anterior muscle of mice treated with cisplatin and/or ZA (*n* = 4–5). Scale bar: 100 μm. Whole body grip strength (reported as peak force) was measured at day 1 and day 14 by taking advantage of a grip strength meter and expressed as the average of the three top pulls from each animal **(C)**. Data are shown as means ± SEM. Significance of the differences: *p < 0.05, ***p < 0.001 vs. V; ^$^*p* < 0.05, ^$$^*p* < 0.01 vs. C; ^&&^*p* < 0.01 vs. ZA.

**Figure 11:**
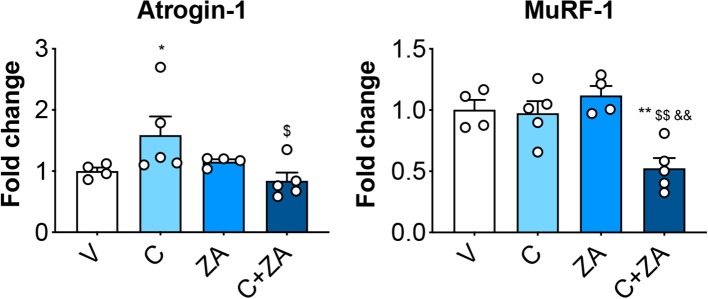
Muscle protein hypercatabolism is counteracted by ZA administration in C-treated mice. mRNA expression for the ubiquitin ligases Atrogin-1 and MuRF-1 in the quadriceps muscle of mice treated with cisplatin and/or ZA (*n* = 4–5). Data are shown as means ± SEM. Significance of the differences: *p < 0.05, **p < 0.01 vs. V; ^$^*p* < 0.05, ^$$^*p* < 0.01 vs. C; ^&&^*p* < 0.01 vs. ZA.

In order to verify whether preservation of bone structure also resulted in protection of muscle mass in animals exposed to chemotherapy, skeletal (tibialis anterior, gastrocnemius and quadriceps) and cardiac muscles were excised from animals administered cisplatin, alone or in combination with ZA (Figure 9). In line with previous findings and our initial results (Figure 2) (37), cisplatin treatment caused significant loss of skeletal muscle mass (Figure 9). Notably, also the heart was significantly smaller in the cisplatin-treated mice (−21%, *p* < 0.001 vs. V) (Figure 9). On the other hand, while ZA alone did not show any direct effects on muscle mass, the combination C+ZA revealed improved muscle size, as suggested by the protection of the tibialis anterior (+16%, *p* < 0.01 vs. C; interaction: *p* < 0.05) and quadriceps (+12%, *p* < 0.05 vs. C), and by the partial preservation of gastrocnemius (+7%, *p* < 0.05 vs. C) and heart weights (+9%, *p* < 0.05 vs. C) (Figure 9). Consistent with the effects on muscle mass, muscle fiber size was also partially preserved in the cisplatin-treated animals receiving ZA, as shown by the quantification of the muscle cross-sectional area (+17%, *p* < 0.05 vs. C) (Figures 10A,B). Interestingly, the ZA-associated protection of muscle mass was also accompanied by substantially preserved muscle strength in the C+ZA group (*p* < 0.001 vs. C), which was 42% higher than the C-treated animals on day 14 (−25%, *p* < 0.01 vs. V; interaction: *p* < 0.01) (Figure 10C). In line with these findings, we investigated the mRNA levels for Atrogin-1 and MuRF-1, ubiquitin ligases normally overexpressed in skeletal muscle during cachexia (40, 43). Atrogin-1 was significantly increased following cisplatin treatment (+54%, *p* < 0.05 vs. V), whereas its expression was returned to control values following ZA administration (Figure 11). On the other hand, MuRF-1 muscle levels were reduced in the mice receiving the combination C+ZA (−48%, *p* < 0.05 vs. V), whereas we did not observe changes in the other experimental groups (Figure 11).

## Discussion

Musculoskeletal derangements are among the most common and most distressing symptoms associated with cancer and its treatment (44, 45), affecting 70–100% of patients receiving chemo-radiation therapies (46–48). Cancer treatments are frequently responsible for a decline of muscle and bone function and the development of muscle weakness and bone frailty, together well-known features of cachexia (49–51). This is a condition frequently observed in upwards of 80% of advanced cancer patients and mainly associated with striking loss of body weight and lean body mass, along with worsening of the quality of life and increased morbidity and mortality rates (52). More importantly, the functional deficits due to muscle weakness have been shown to persist for months to years following remission (53–56), thereby causing a significant worsening of the quality of life (57). Unfortunately, cancer-related muscle weakness is further intensified with the aggressiveness of chemotherapy, and no treatments have been shown to relieve such conditions thus far (58, 59).

In line with data from Chen et al. (37), here we showed that cisplatin, a platinum-based alkylating agent usually prescribed for the treatment of solid tumors, leads to severe musculoskeletal deficits in growing mice. Consistent with previous findings (60), our observations generated in an *in vitro* model also suggest that chemotherapy-dependent effects on muscle fiber size are triggered by circulating factors, likely released upon bone destruction. We previously reported that chronic administration of Folfiri, a chemotherapy regimen often prescribed for the treatment of solid tumors, participates in the pathogenesis of cachexia by affecting muscle mass and function and by causing dramatic loss of trabecular bone (9, 13). In line with our observations, Hain et al. (21) recently showed that treatment with another platinum-based agent, carboplatin, despite being effective in counteracting tumor dissemination in a model of metastatic breast cancer, contributes to significant muscle atrophy and weakness, also accompanied by loss of trabecular bone.

The correlation between chemotherapy treatment and decreases in bone mass primarily due to the negative effects of anticancer drugs on bone remodeling has been investigated for quite some time (61, 62). For example, imatinib, used for the treatment of gastrointestinal tumors and leukemias, was found to directly target platelet-derived growth factor (PDGF) receptor, among others (63). Similarly, taxanes were shown to cause myelosuppression and, in turn, bone loss and increased levels of inflammatory mediators (64). Methotrexate, routinely prescribed for the treatment of several solid tumors, was reported to directly target bone tissue and promote bone degeneration by increasing the expression of IL-6 and IL-11 (65). Further, corticosteroids, frequently used in combination with anticancer agents, were shown to directly affect bone mass by reducing osteoblast differentiation and by increasing osteoclast-dependent bone resorption (66). At the same time, chemotherapy was also reported to cause bone damage by impinging on indirect systemic effects involving gonadal suppression. Indeed, cytotoxic chemotherapy was recently associated with significant gonadal damage (67, 68). Similarly, ovarian failure resulting from chemotherapy treatments in premenopausal women with breast cancer was shown to promote rapid bone loss (69). Interestingly, hypogonadism in patients receiving anticancer treatments was also linked to the occurrence of musculoskeletal abnormalities (70, 71). Nevertheless, the mechanism(s) responsible for changes in bone mass and relationship to changes in muscle homeostasis, especially in a setting of chemotherapy treatment, is not completely understood.

In the present study, we administered cisplatin in combination with zoledronic acid, a bone-targeted drug used to preserve bone mass in osteoporosis, to investigate whether the treatment with antiresorptive drugs was able to preserve bone mass, as well as muscle size and strength following chemotherapy administration. As shown in our experimental results, zoledronic acid was able to completely preserve cancellous and cortical bone loss in mice receiving cisplatin, without apparent signs of toxicity. Notably, zoledronic acid proved effective in partially preserving muscle mass and strength, thereby supporting the concept that bone-derived factors play a role in the muscle deficits in cachexia induced by chemotherapy. We can speculate that the absence of a complete protective effect of zoledronic acid on muscle mass may be due to the combined action of other non-bone derived mediators, and/or direct toxicity of chemotherapy on the muscle fibers. However, whether zoledronic acid is also able to affect the levels of bone-derived factors remains unknown.

Of note, the improvement in muscle mass and muscle strength was also accompanied by a normalization of the levels of Atrogin-1, a ubiquitin ligase normally upregulated in conditions characterized by skeletal muscle atrophy (43), also suggesting that enhanced muscle hypercatabolism could play a role in cisplatin-induced muscle wasting. On the other hand, MuRF-1, previously shown significantly elevated in the skeletal muscle of cachectic mice (40), was significantly downregulated in the animals receiving the combination treatment with respect to the control, consistent with data reporting protection of muscle mass in MuRF-1 knock-out mice exposed to the pro-catabolic drug dexamethasone (72). On the other hand, cisplatin alone did not cause any upregulation, at least at the performed time point. In this regard, our observation that zoledronic acid was unable to alter skeletal muscle wet weights in normal conditions, but rather only in combination with chemotherapy, further supports the concept that abnormal muscle-bone interactions may play a role in the pathogenesis of cachexia.

Recently, major interest has grown toward understanding the so-called “muscle-bone crosstalk.” According to this concept, muscle tissue represents a storehouse of “myokines,” known to affect bone mass by regulating bone destruction and bone formation (16). Conversely, bone-secreted factors would seem to influence skeletal muscle beyond the mechanical relationship in loading and primarily through the release of soluble mediators (known as “osteokines”) (73, 74). These are now known to directly influence muscle mass by contributing to regulation of size and contractility (15–18). Exacerbated loss of bone along with increased osteolysis are well-documented in breast cancers and multiple myeloma, along with formation of metastases to bone (19, 75). In particular, Waning *et al*. previously suggested that enhanced bone resorption associated with cancer dissemination results in release of TGFβ from the bone matrix, which in turn causes modulation of muscle regulatory pathways and contributes to muscle weakness (19). Similarly, investigative efforts from our group have shown that bone loss also occurs in the absence of bone metastases and frequently associates with changes of muscle homeostasis and function (38, 76), although the causative mechanism(s) responsible for such abnormalities have not been completely elucidated.

In this regard, pro-inflammatory cytokines, such as TGFβ-family ligands, are known to play a role in the regulation of skeletal muscle mass (77). These cytokines were shown to be released from the mineralized matrix in conditions associated with bone destruction, including cachexia (19, 36, 78, 79). This is also in line with previous evidence from our group, showing that administration of an activin receptor type-2B (ACVR2B) antagonist, previously shown to improve muscle mass and prolong survival in tumor-bearing mice (80), was able to completely restore muscle and bone mass in animals chronically administered Folfiri (13). Therefore, our results suggest that cessation of basal homeostatic skeletal turnover alone is not directly involved in the regulation of skeletal muscle mass, whereas bisphosphonate-induced correction of abnormal bone resorption in cachexia may be sufficient to ameliorate skeletal muscle atrophy *per se*.

Interestingly, Børsheim et al. (35) previously reported that children affected with unintentional burn-injury not only show preservation of bone mass upon treatment with bisphosphonates, but also significant improvements in muscle mass. In a recent collaborative investigation, we provided evidence supporting the idea that TGFβ plays a pivotal role in causing muscle atrophy in burn children, usually characterized by dramatic loss of bone and muscle mass, whereas administration of the bone-protecting agent pamidronate would seem to improve muscle size by counteracting the TGFβ-dependent signaling and restoring the proper muscle anabolism (36). However, it remains unclear whether similar mechanisms also participate in causing bone loss and muscle wasting and whether preservation of bone mass effectively improves muscle size and function following chemotherapy administration.

The idea that bone and muscle communicate at a biochemical level by exchanging soluble mediators also provides new avenues for direct pharmacological interventions aimed at targeting these factors, as reviewed by Brotto and Bonewald (16). In particular, bone-targeted agents, primarily bisphosphonates, are potent antiresorptive drugs, routinely used in the clinic for the treatment of post-menopausal osteoporosis and bone frailty associated with chronic conditions or metastatic cancers (22). These drugs were originally designed to counteract osteoclast activity and bone resorption, and subsequently shown to prevent the release of pro-inflammatory cytokines and other signaling molecules, including TGFβ, BMP2, and IGF-1, from the bone matrix (78, 79, 81, 82). In adult individuals, altered bone “coupling,” i.e., the physiologic coordination of bone resorption with bone formation (83), often occurs with increases in osteoclast activity with little to no change in osteoblast-driven bone formation. This results in an imbalance in bone remodeling and leads to decreased bone mass, increased risk for fractures and worsened survival rates (84). We and others have provided evidence of bone loss in both cancer- and chemotherapy-induced cachexia, likely suggesting an imbalance in osteoclast vs. osteoblast activity in association with loss of muscle mass and strength (13, 21, 36, 38, 76).

Notably, bone is now often referred to as an endocrine organ, secreting osteogenic factors (i.e., osteokines), which can be released during resorption (16). Originally, all these factors were thought to mainly take part to the regulation of bone mass, although it is becoming clear that these osteokines can also affect muscle homeostasis (85). For example, while components of the Wnt/®β-catenin pathway are important regulators of bone mass, it has been shown that Wnts also affect muscle by supporting myogenesis and muscle function (86). In a similar manner, receptor activator of nuclear factor kappa-B ligand (RANKL) and its natural decoy receptor osteoprotegerin (OPG) are mainly produced by bone cells and are critical for the activation of osteoclasts and the regulation of bone resorption (87). Interestingly, the receptor for RANKL, known as RANK, is also expressed in skeletal muscle, where it appears to regulate muscle contractility (88), whereas anti-RANKL antibodies and OPG-Fc have been shown to improve muscle size and function in dystrophic *mdx* mice (89). Another example is provided by osteocalcin, mainly produced by mature osteoblasts and osteocytes. Osteocalcin not only was shown to regulate glucose and energy metabolism, as well as fertility in male mice and ectopic calcification, but also appears to affect muscle mass, based on the evidence that supplementation with osteocalcin restores reduced exercise capacity in mice and improves muscle strength (90). Moreover, elevated levels of the osteocyte-derived fibroblast growth factor 23 (FGF23) were also shown to negatively impact cardiac muscle by increasing the risk of heart disease, left ventricular hypertrophy, vascular calcification, although no effects were conclusively described in skeletal muscle (91). The osteocyte factor prostaglandin E2 (PGE2), normally released in response to fluid flow shear stress, was also shown to affect muscle growth and function by acting as a potent stimulator of myogenic differentiation in primary myoblasts/myotubes (92). Interestingly, osteoclasts were shown to secrete soluble factors endowed with muscle-protective properties. This is the case of cardiotrophin-1 (CT-1) and sphingosine-1-phosphate (S1P) (93). Specifically, CT-1, an IL-6 superfamily member signaling through binding to the leukemia inhibitory factor (LIF) receptor, was described as an osteoclast-derived factor critically involved in bone remodeling (94). However, recent observations suggest that CT-1 may also directly affect muscle tissue. Indeed, CT-1 was shown to exert cardioprotective effects and to stimulate myogenic and vascular remodeling of the heart, as well as to increase extraocular muscle mass and strength in experimental animal models (95–97). Similarly, S1P, a bioactive lipid that acts via G protein-coupled receptors previously implicated in several osteogenesis-related processes, including differentiation and survival of osteoblasts and their subsequent coupling with osteoclasts (98), was also shown to positively impact muscle tissue, through the regulation of skeletal myoblast proliferation (99), as well as in the control of normal cardiac development (100) and of smooth muscle cell proliferation, migration and contraction (101). In our study we did not assess the levels of these factors, although the evidence that muscle mass was improved by administration of an anti-resorptive agent, such as zoledronate, appears to suggest that some of these osteokines, normally released from the bone matrix upon activation of bone resorption, may well play a role in the regulation of muscle mass in cachexia induced by chemotherapy.

A strength of our approach is that we did circumvent the lack of muscle targeted therapeutics and, instead, aimed at repurposing existing, FDA-approved drugs for alternate uses. While the long-term side effects of bisphosphonate treatment have been previously characterized (102), whether bisphosphonates also promote long-term toxicities on skeletal muscle, especially in subjects treated with chemotherapy, remain to be clarified, along with the impact that these drugs may have on efficacy and tolerability of anticancer drugs. Our data may appear in disagreement with previous studies reporting no effects or moderate toxicities associated with long-term (>3 years) bisphosphonate administration on skeletal muscle mass in post-menopausal osteoporotic women, showing bone and muscle defects at time of first treatment (18, 103). However, our experimental model, using normal, healthy animals exposed to chemotherapy and treated with bisphosphonates for the entire duration of the experiment (i.e., since day 1) is not directly comparable with such studies. Taking into account also the results reported by Børsheim et al. (35), showing beneficial effects on muscle mass in pediatric patients administered pamidronate shortly after burn injury, our observations support the idea that bisphosphonates, rather than rescue muscle mass once musculoskeletal complications are already established, may instead contribute to preserve skeletal muscle in conditions normally associated with pro-cachectic stimuli. Moreover, it is important to point out that our studies were conducted in young, skeletally immature growing animals, therefore characterized by elevated rates of bone formation, as also supported by the analysis of the bone histomorphometry data. At this time, we cannot exclude that different outcomes might occur by using the same therapeutic approach in adult or aged animals, in accordance with evidence suggesting that bisphosphonates concomitantly impair bone formation in adult individuals, thus increasing the likelihood of long-term adverse events (104).

Nonetheless, the use of bisphosphonates as anticancer agents has recently been investigated due to their beneficial properties in counteracting the formation of bone metastases and preventing adverse skeletal events in cancer (24, 25, 33). In this study, we did not take into examination animals bearing cancers plus chemotherapy. Although this choice may represent a limitation of our study, in this pre-clinical investigation we decided to focus on establishing mechanisms responsible for chemotherapy induced muscle wasting. In this regard, the findings described here corroborate the idea that chemotherapy-associated toxicities negatively impact bone and muscle mass, thus leading to phenotypes consistent with cachexia.

In this study we focused on investigating the musculoskeletal abnormalities that occur following chemotherapy treatment, with the ultimate goal of determining whether anti-resorptive drugs could contribute to preserve both bone and muscle in a setting of anticancer treatment. In this regard, we have to keep in mind that the dosing for cisplatin and zoledronate used in our experimental model, tested in previous studies (37, 60), does not necessarily compare to the one usually prescribed for humans. Indeed, by converting the animal dosing to the human equivalent dose, calculated following the guidelines reported in Nair and Jacob (105), our animals may appear underdosed, especially if compared to the clinical setting (106, 107). However, it is important to note that as patients are normally treated with either drugs once every 2-to-4 weeks, on the contrary our experimental animals received multiple treatments over 2 weeks.

In conclusion, in the present study we provide evidence that bone-protecting agents, such as bisphosphonates, may be combined with routinely used anticancer drugs to the extent of reducing their associated toxicities and, ultimately, mitigating the occurrence of chemotherapy-associated musculoskeletal abnormalities. Moreover, our experimental data corroborate the possibility that bisphosphonates are administered in combination with chemotherapeutics since first treatment in order to maximize their efficacy in preserving muscle and bone. Overall, we expect our findings will pave the way to major investigations on the use of bisphosphonates in oncology care and encourage future studies aimed at defining zoledronic acid as a new anti-cachexia treatment in combination with traditional chemotherapy or cancer.

## Data Availability Statement

The datasets generated for this study are available on request to the corresponding author.

## Ethics Statement

The animal study was reviewed and approved by Institutional Animal Care and Use Committee at Indiana University School of Medicine.

## Author Contributions

AE, FP, and AB conceived and designed the experiments. AE, FP, JH, and AB performed the *in vitro* and *in vivo* experiments, the body composition assessment, the muscle function analysis, the microCT analysis of the bone, and the molecular characterization of cachexia. LB and LP provided support for the bone studies. AE, FP, LB, LP, and AB wrote and edited the paper.

### Conflict of Interest

The authors declare that the research was conducted in the absence of any commercial or financial relationships that could be construed as a potential conflict of interest.
