# Supplementary material for: Bisphosphonate Treatment Ameliorates Chemotherapy-Induced Bone and Muscle Abnormalities in Young Mice
Source: Front Endocrinol (Lausanne). 2019 Nov 19;10:809. doi: 10.3389/fendo.2019.00809 (PMC6877551; doi:10.3389/fendo.2019.00809)
Supplement: Supplementary file 1 [file Data_Sheet_1.PDF]

Figure S1

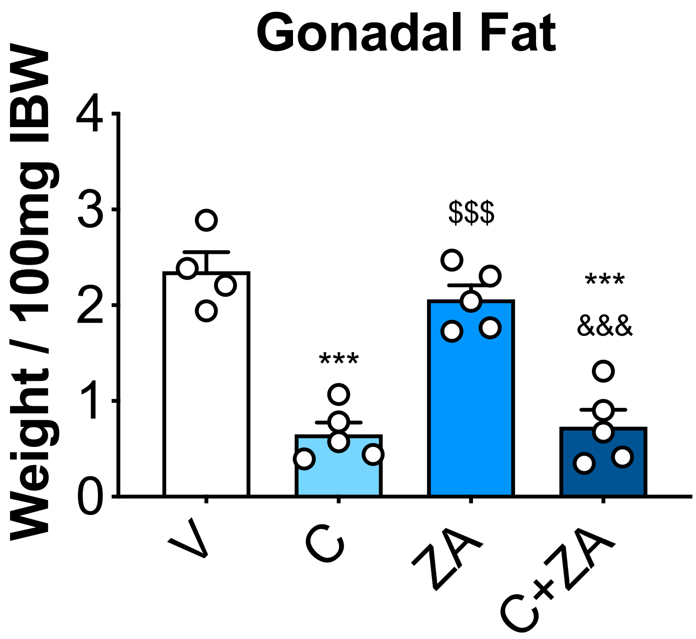

Cisplatin causes severe loss of adipose tissue, which is not preserved by ZA treatment. Epididymal (gonadal) adipose tissue in mice receiving V, C, ZA and C+ZA (n=4-5). Weights were normalized to the Initial Body Weight (IBW) and expressed as weight/100mg IBW. Data are expressed as means ± SD. Significance of the differences: $^{***}p<0.001$ *vs.* V; $^{\$\$\$}p<0.001$ *vs.* C; $^{\&\&\&}p<0.001$ *vs.* ZA.
